# Suggesting a Way to Understand the Actual Potential of Anti-Alzheimer’s Disease Drugs That Show Promise in Transgenic Mouse Models

**DOI:** 10.3389/fneur.2015.00206

**Published:** 2015-10-06

**Authors:** Rafael Franco, Milos Petrovic

**Affiliations:** ^1^Departament de Bioquímica i Biologia Molecular, Facultat de Biologia, Universitat de Barcelona, Barcelona, Spain; ^2^Centro de Investigación Biomédica en Red: Enfermedades Neurodegenerativas (CIBERNED), Instituto de Salud Carlos III, Madrid, Spain; ^3^School of Pharmacy and Biomedical Sciences, University of Central Lancashire, Preston, UK

**Keywords:** chiral drug, memory, Alzheimer’s disease, nootropic, neuroprotection, enantiomers

One conundrum in Alzheimer’s disease (AD) research using transgenic mouse models is the high amount of successful memory-enhancing drugs. By contrast, very few drugs and of limited efficacy are available for humans having this pathology. As previously discussed (1), the advance in this field, i.e., to fulfill the translational facet of anti-AD research, requires deciphering why so many different drugs (or therapeutic interventions, such as exercise or training) have memory-enhancing properties in transgenic models of the disease. Transgenic animals do not accurately reflect the human disease, as they overexpress proteins with mutations that appear only in a reduced percentage of patients (2). The majority of patients have late-onset clinical symptoms due to multiple factors many of which may be circumstantial. On waiting for the development of novel animals models that may, eventually, shorten the distance between the lab bench and the bedside (3), we should take advantage of the huge amount of data showing promise of different drugs in transgenic models. A way to do it is by designing medium-to-high throughput experiments to compare anti-AD effects of closely related drugs. In this commentary, we focus on small drugs with the same chemical formula, but with different 3-D structure.

A significant number of drugs approved for human consumption for fairly different illnesses have a special structural characteristic called stereoisomerism (see Figure 1). Examples of small molecules with alternative stereoisomer variants (enantiomers) that are already marketed for human consumption include verapamil, ibuprofen, citalopram, and thalidomide. Due to the difficulties in isolating the two enantiomer species and other operational reasons, mixtures of the two species are approved for human consumption.

**Figure 1:**
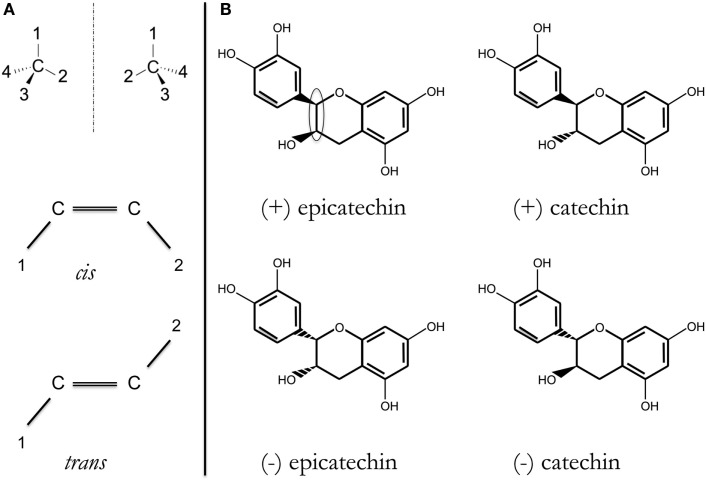
**(A)** Stereoisomers have the same formula and the same bonds, but different 3-D structure. Two of the most common are as follows: R/S (D/L, ± ) enantiomers or *cis*/*trans* isomers, depending on configuration around, respectively, an asymmetric carbon (top) or a double C = C bond (bottom). Substituents are indicated by 1, 2, 3, or 4. In each case, two different compounds may exist; more if there is a combination of asymmetric carbons and/or double C =C bonds. **(B)** Structure of the four possible catechin/epicatechin stereoisomers resulting from substituent configuration around two contiguous asymmetric carbons [within the ellipsoid in (+)-epicatechin structure]. It should be noted that the structures of these four compounds are dissimilar in different databases (not only in Wikipedia but also in those targeting more expert audience, e.g., chemistry-related or commercial vendor databases).

## Verapamil

Verapamil, the calcium channel blocker indicated in a variety of cardiovascular ailments, is the mixture of two enantiomers: (±)-2-(3,4-dimethoxyphenyl)-5-{[2-(3,4-dimethoxyphenyl)ethyl]-(methyl)amino}-2-prop-2-ylpentanenitrile. Despite the higher efficacy of the *levo* isomer, already reported in 1985 for atrioventricular conduction (4), the drug is still marketed as a mixture of *levo* and *dextro* species. The potential of verapamil in AD was assayed 18 years ago under the strategy of discovering new uses for drugs already approved for human consumption. As verapamil ameliorates cognitive and non-cognitive deficits (5), we propose to test separately the *levo* and *dextro* verapamil molecules in AD models.

## Ibuprofen

Controversy surrounds the usefulness of anti-inflammatory drugs in AD and one example is provided by ibuprofen, which suppresses plaque pathology in the Tg2576 AD model (6), while not improving deficits in the 5XFAD AD model (7). To our knowledge, the two components of ibuprofen [(±)2-(4-Isobutylphenyl)propanoic acid] have not been tested separately.

## Thalidomide

Thalidomide, the leprostatic and sedative drug with cognition-enhancement and anti-amyloid β (Aβ) properties in mice models (8, 9), is constituted of two enantiomers [(±)-2-(2,6-dioxo-3-piperidinyl)-1H-isoindole-1,3(2H)-dione] that have not been individually tested in AD models.

Would it be convenient to test the different stereoisomers individually in AD models? Indeed, living animals are asymmetric at the macroscopic and at the molecular level (e.g., mammalian proteins are built up of l-, but not d-amino acids), and, therefore, it is predictable that one stereoisomer may be more efficacious than the other(s).

Two enantiomers will likely have differential pharmacokinetics, differential metabolism, and differential mode of action (see Ref. (10) for recent review). Therefore, it is improbable that two enantiomers have similar – beneficial – effects in AD models, otherwise their action would be a general chemical one (for instance anti-oxidant, as discussed below) and not due to a specific mode of action. If the mode of action of a given asymmetric drug is specific, i.e., via inhibiting an enzyme or interacting with a receptor, chances of stereoisomers having similar *in vivo* potency are scarce. In that sense, one enantiomer may be the negative control of the other. One further concern in validating data from transgenic AD models is the usual finding of multiple beneficial effects of a given drug (e.g., improving spatial memory, decreasing Aβ burden, reducing tau hyperphosphorylation). A compound having many benefits should be the exception and not the rule. Accordingly, scientists may consider which property is under study (behavioral, biochemical, etc.) and select the appropriate experimental model. It would also be desirable to determine the pharmacokinetics of the promising drugs to establish whether the individual enantiomers reach the brain at physiologically relevant concentrations or not

## Citalopram

Escitalopram is one of the few examples of single stereoisomers that have reached the market. Citalopram (sold in different countries as: Celexa, Seropram, Talpram, Prisdal, Zentius, Cipramil, or generic citalopram) is one of the best-seller CNS drugs. Citalopram contains the racemic mixture of R/S (or ±) 1-[3-(dimethylamino)propyl]-1-(4-fluorophenyl)-3H-2-benzofuran-5-carbonitrile, but the S-enantiomer is better in inhibiting serotonin uptake and providing anxiolytic and anti-depressant effects. Isolation of the S molecule led to the approval of escitalopram for clinical use (sold as Lexapro, Cipralex, or generic escitalopram). The difference between enantiomers is not a trivial one, as health and socio-economic benefits of escitalopram versus citalopram have been substantiated (11–14). Citalopram has been used in the Citalopram for Agitation in Alzheimer Disease Study (CitAD) randomized clinical with promising results (15). Thus, it would be reasonable to undertake another clinical trial to compare the effects of citalopram versus escitalopram in AD patients. Complementarily, it would be convenient to compare in animal AD models the efficacy of the two stereoisomers of citalopram in a variety of cognitive and molecular read-outs.

## Catechins

Recently, substances with anti-oxidant properties have been found to be neuro-protective, indicating them as potential tools to combat AD. Polyphenols derived from plants and used in human nutrition may have anti-oxidant properties and neuro-protective potential. Let us consider close plant-derived compounds, tested in different experimental systems: (+)-catechin and (−)-epicatechin. One recent report has shown promise of (−)-epicatechin in transgenic AD models (16). However, *in vitro*, (+)-catechin and (−)-epicatechin are equally efficacious in inhibiting formation of Aβ fibrils from the precursor peptides (Aβ_1–40_ or Aβ_1–42_) (17). Is the anti-oxidant property of these compounds also responsible for the anti-AD effects of (−)-epicatechin in transgenic animals? Otherwise, how may the two stereoisomers have similar efficacy? A good negative control is needed that should be as similar as possible in all experimental set-ups. As commented earlier, one good possibility is taking advantage of enantiomers/stereoisomers. Catechin and epicatechin have identical formula, C_15_H_14_O_6_, with four structural possibilities, namely four diastereoisomers. If all four of them act as anti-oxidants, all should display similar efficacy. If they are, however, acting by a specific mechanism, they should have significantly different efficacies. Catechin has a *trans* configuration and epicatechin has a *cis* configuration, and each of them has a (+) enantiomer and a (−) enantiomer (Figure 1). We find the possibility of testing these four molecules in equal AD models, at the same time and by the same experimenter and in a blind way (ideally double blind) of high added value. By providing robust modes of action, the differential effect of stereoisomers in AD models should help in accelerating translational anti-AD research.

## Conflict of Interest Statement

The authors declare that the research was conducted in the absence of any commercial or financial relationships that could be construed as a potential conflict of interest.
